# Osteoporosis associated vertebral fractures—Health economic implications

**DOI:** 10.1371/journal.pone.0178209

**Published:** 2017-05-22

**Authors:** Julian Joestl, Nikolaus Lang, Adam Bukaty, Thomas M. Tiefenboeck, Patrick Platzer

**Affiliations:** 1 Department of Trauma Surgery, Medical University of Vienna, Austria; 2 Division of General Anaesthesia and Intensive Care Medicine, Medical University of Vienna, Austria; Georgia Regents University, UNITED STATES

## Abstract

**Introduction:**

Osteoporosis-associated vertebral fractures represent an increasing clinical and public health problem, one with important socioeconomic effects within western countries.

The purpose of this study was to analyse demographic, medical, gender and socioeconomic aspects of osteoporotic vertebral fractures of the thoracic or lumbar spine over a period of at least 10-years.

**Material and methods:**

Included for analysis were 694 patients who had suffered a vertebral fracture due to primary or secondary osteoporosis, and who were treated at our Level-I trauma center between 2000 and 2013. Collected data included demographic, medical and socioeconomic aspects.

**Results:**

Clinical results revealed that 669 patients (96%) were treated conservatively. The remaining 25 patients (4%) underwent surgical therapy: 4 were treated with vertebroplasty, 15 with kyphoplasty and 6 patients with posterior stabilization. The mean age was 75.6 years (range: 50–98), with the vast majority of patients being female (n = 515). A statistically significant demographic difference (i.e., increase) in fractures was observed between the age groups 60–69 and 70–79 (p = 0.041). Concerning socioeconomic aspects, statistical analysis showed that the number of sick leaves and the need for professional domestic help was higher in female patients. Concerning treatment costs, statistical analysis did not reveal any significant differences between female and male patients.

**Conclusion:**

Significant gender differences–to the detriment of the female population–could be demonstrated within this study. A regrettably low rate of adequate treatment after diagnosis of osteoporosis and its associated fractures–specifically relating to primary and secondary prevention–could also be identified. To prospectively avoid complications and consequential cost increases, more awareness of the necessity for prevention, early diagnosis and adequate treatment of osteoporosis and its related fractures should be considered.

## Introduction

Osteoporosis has become a major public health problem, particularly in industrial nations. Consequently, gender differences in osteoporosis and its related fractures have been garnering increasing attention [1]. Statistical compilations reveal that nearly every third woman, and every tenth man, suffer from osteoporosis [2]. However, the medical–and particularly the socioeconomic–burden of osteoporosis-related fractures are notably higher in men [2–4]. The current literature reveals that more than 60% of patients with osteoporosis sustain an associated fracture once in their lifetime. Vertebral compression fractures in the thoracic or lumbar spine are the most common type of osteoporotic fractures, accounting for almost as many fractures as hip and distal radius fractures combined. This fact ultimately leads to a dramatic increase in financial and human costs [2, 3, 5].

The purpose of this study was to analyse demographic, medical, gender and socioeconomic aspects of 694 osteoporosis-associated vertebral fractures at the thoracic or lumbar spine over a period of at least 10-years.

## Materials and methods

The authors performed a review of their Level-I trauma center’s trauma database–a prospectively gathered database established for the registration of injury characteristics (e.g., type, mechanism, e.g.) and the demographic data of trauma patients. We identified 694 patients who had suffered a fracture caused by either primary or secondary osteoporosis, and who had been treated at the department of trauma surgery between 2000 and 2013. The study was approved by our Institutional Review Board (Ethics Committee of the Medical University of Vienna, registration number 2011/896) and was registered in a publicly accessible registry (clinicaltrials.gov ID: NCT02386865).

All collected datasets were finally reviewed against the inclusion criteria: Patients must have been older than 50 years of age, and must have presented with either vertebral fractures of the thoracic or lumbar spine caused by a low-energy trauma or without any trauma, or with vertebral fractures of the thoracic or lumbar spine with typical osteoporotic deformity of the vertebrae (i.e., wedge, fish, or flat).

Exclusion criteria were defined as: Patients younger than 50 years of age; patients with vertebral fractures at the cervical spine; patients with vertebral fractures at the thoracic or lumbar spine caused by a high-energy trauma (e.g., motor-vehicle accident, sports-related accident, fall from a considerable height); patients suffering from a malignancy, in whom a pathological fracture could be presumed; patients whose dataset of follow-up monitoring was lacking, thus making an evaluation of osteoporotic involvement impossible.

The collected data was relatively complete due to the healthcare and data collecting system in Austria. Data was assessed and compared with respect to (1) the patients’ age and gender distribution, (2) their diagnoses, treatments and outcomes–as well as to (3) the prevention of osteoporosis and its associated fractures, the burden of osteoporotic fractures in one’s daily routine, and the treatment costs to the health care system.

Treatment costs were calculated using a “procedure-oriented health care financing” of health care providers in the author’s home country (Austria).

### Statistics

For descriptive statistics, an age classification of the included patients was performed. In order to determine potential differences among various age categories, and to allow a better illustration concerning the distribution of age characteristics, 5 groups were created: (1) 50–59 years of age; (2) 60–69 years of age; (3) 70–79 years of age; (4) 80–89 years of age; (5) over 90 years of age.

For univariate analysis of the data, a chi-square test was performed to determine statistically significant differences in direct comparison of categorical variables, with special emphasis on gender-related deviations. A p-value ≤0.05 was used for interpretation.

For multivariate analysis, we performed a logistic regression analysis with a 95% confidence interval. Regression coefficients were calculated for the interpretation and description of statistical results. Treatment outcome was used as the dependent variable for regression, whereas age, gender, methods of treatment, neurological disorders, rehabilitation, duration of hospital stay, professional domestic help, prevention of osteoporosis and treatment costs posed as corresponding variables.

## Results

### Demographic data

694 patients met the inclusion criteria and were finally enrolled in this study. The patients exhibited an average age of 75.6 years (range: 50–98 years), with the overwhelming majority being female (n = 515). The sex distribution of patients with osteoporotic fractures is shown in Table 1. Statistical analysis revealed that, in all age groups, a significantly larger proportion of included patients with osteoporotic vertebral fractures were women (p <0.05). Men only represented between 18% (in the age group 80–89) and 33% (in the age group 70–79) of the patients.

**Table 1:** Osteoporotic fractures. Distribution of patients with osteoporotic fractures.

Age Group	Patients with Osteoporotic Vertebral Body Fractures	Female	Male	Gender Differences (p-value)
50–59	73	49 (67%)	24 (33%)	p = 0.043
60–69	139	102 (73%)	37 (27%)	p = 0.025
70–79	185	121 (65%)	64 (35%)	p = 0.048
80–89	219	180 (82%)	39 (18%)	p = 0.012
Over 90	78	63 (81%)	15 (19%)	p = 0.028
All Age Groups	694	515 (74%)	179 (26%)	p = 0.035

### Patient history

480 patients (69%) consulted the outpatient clinic because of pain in the either the thoracic and lumbar spine, or the thoraco-lumbar and lumbo-sacral junction. 32 (5%) reported no back pain. In 182 cases (26%), the history provided no information on pain. Clinical records indicated several mechanisms of injury. 262 injuries (38%) resulted from falls in one’s own home, 85 (12%) individuals had fractures from falls in their care retirement homes and 51 cases (7%) occurred in other settings. In 177 patients (25%), no information was provided as to the site of the fall. Both sudden pain during the lifting of heavy objects (n = 16; 2%) and pain without any trauma (n = 19; 3%) were also reported. 27 patients (4%) suffered from some other low-energy trauma, while in 57 patients (8%), no information could be obtained as to the cause of trauma.

### Physical examination

12 patients (1.5%) exhibited hematomas, swelling, abrasions or contusion marks at the height of the affected vertebral segment. Elderly patients displayed a higher percentage of external injuries to other body parts (n = 186; 27%), assumedly due to their diminished reactivity and postural control. 337 patients (49%) reported to the outpatient clinic on the same day as the injury, 133 patients (19%) on either the next day or two days after the injury. 53 patients (8%) showed up between the third and seventh day, while 139 (19%) came in more than 7 days after the injury. In 32 cases (5%), no information was reported as to the time of accident. 541 patients (78%) came in for a primary treatment (PT) and 116 (17%) for a consecutive treatment (CT) because initial therapy had not been successful. 14 patients (2%) were transferred from another hospital (Tf), 11 (1%) consulted the outpatient clinic for a follow-up exam (FE) and 12 (2%) presented themselves for a long-term exam (LTE) after therapy had already been concluded. The number of patients with neurological loss of function due to vertebral body fractures was low (n = 23; 3%). Sensory, motor or circulatory impairing symptoms (S/M/C) of the extremities were reported in only 13 patients (2%). Patients were categorized according to potential risk factors for osteoporosis: 44 patients (6%) exhibited alcohol abuse (more than 2 units per day), 81 patients (12%) tobacco use, 138 (20%) exhibited a lack of physical activity and 34 (5%) had a very low body weight. 385 patients (55%) took medications which cause osteoporosis and 111 patients (16%) suffered from diseases which result in bone loss. In total, 529 patients (76%) displayed a risk factor for osteoporosis. Statistical analysis revealed that alcohol abuse and smoking were seen more frequently in men than in women; all other risk factors did not show any significant differences. When asked about alcohol consumption, 185 patients (26%) claimed not to drink any alcohol, 91 (13%) drank sometimes (less than 1 unit per day), 25 (4%) drank regularly (more than 2 units per day) and 19 (3%) were addicted to alcohol. In 374 cases (54%), no statement concerning alcohol consumption was given. 220 patients (32%) were recorded as being non-smokers, while 19 (3%) were former smokers who had quit. 62 patients (9%) claimed to be active smokers. In 393 cases (56%), no information was obtained concerning smoking habits. A lack of physical activity due to walking handicaps (e.g., the use of walking aids) or impairments (e.g., hemiparesis after an insult) was documented in 138 patients (20%). Other age-related problems were not obtained from the available history. In order to assess the risk factor “low body weight”, patients were separated into the categories “underweight” (<18.5 BMI), “normal weight” (18.5–25 BMI), “overweight” (25–30 BMI) and “obese” (>30 BMI). According to these categories, 34 patients (5%) were considered to be underweight, 142 (20%) were of normal weight, 39 (6%) were overweight and 24 (3%) were deemed obese. In 455 cases (65%), no information was obtained. In total, 385 patients (55%) took a medication whose major side effect is osteoporosis. 16 (2%) took glucocorticoids, 58 (8%) oral anticoagulation drugs, 94 (13%) heparins, 206 (30%) proton pump inhibitors (PPI) or antacids, 8 (1%) anticonvulsants and 3 patients (0.4%) took calcineurin inhibitors. No patient was receiving highly active antiretroviral therapy (HAART). Aromatase inhibitors and androgen deprivation therapy, which can also cause osteoporosis, were not considered, as carcinoma patients were excluded from this study. 111 patients (16%) suffered from diseases which are known risk factors for osteoporosis. 86 (13%) were endocrinological diseases, 16 (2%) were intestinal diseases (e.g., Crohn’s disease, ulcerative colitis, Sprue, gastrectomy) and 9 patients (1%) suffered from rheumatoid arthritis.

### Imaging

The diagnosis of an osteoporotic vertebral fracture was achieved by means of conventional X-ray in all 694 cases (100%). Additional computed tomography was performed in 198 cases (29%), and in 26 cases (4%) magnetic resonance imaging was obtained to reinforce the diagnosis. Of the 694 included patients, a total of 1042 vertebral body fractures were diagnosed. 237 patients (34%) showed 2 or more osteoporotic vertebral fractures. 320 (31%) of the 1042 fractures were located at the thoracic level, with 722 (69%) being located at the lumbar level the majority of fractures occurred near the thoraco-lumbar junction T9 to L2 (63%). 164 fractures (16%) were wedge-shaped, 107 fractures (10%) had a fish-like deformity and 9 (1%) resulted in flat vertebrae. The remaining 744 osteoporotic vertebral fractures did not exhibit a typical osteoporotic deformity. Relating to vertebral height reduction, in 232 (22%) cases the reduction was minor (grade 1, 20–25%), in 541 cases (52%) moderate (grade 2, 25–40%) and in 178 (17%) cases severe (grade 3, over 40%). The remaining 91 fractures (9%) did not reveal any notable height reduction. Data analysis showed that in 148 patients (21%), an additional osteoporotic fracture was diagnosed. 91 of these patients (13%) exhibited a femoral neck fracture, 31 (4%) a forearm fracture and in 26 patients (3%) rib fractures. A statistically significant increase in osteoporotic vertebral fractures was verified between the age groups 60–69 and 70–79 (p = 0.041).

### Treatment

669 (96%) of the 694 patients with osteoporotic vertebral body fractures were treated conservatively; the remaining 25 patients (4%) underwent surgical therapy.

### Conservative treatment

384 (57%) of the 669 conservatively managed patients were treated as outpatients, and 285 (43%) as inpatients.

Concerning the conservative treatment of outpatients, 366 (95%) of 384 patients received an adequate medicamentous pain therapy. 231 (32%) were additionally prescribed rest and protective measures, 30 (8%) were instructed to begin mobilization with the aid of a general practitioner or nursing home staff (depending on their individual situation) and another 30 cases (8%) were treated with an orthesis. 51 patients (13%) were referred to a general practitioner (GP) for additional pain therapy.

Concerning the conservative treatment of inpatients, 215 (75%) were mobilized under physiotherapeutic supervision. 17 patients (6%) were required to stay in bed–for rest and protection–for a period of 2–8 weeks. 56 patients (30%) were additionally supplied with an orthesis, and 11 (4%) underwent cast immobilization for an average of 12 weeks. 169 patients (47%) received infusions with nonsteroidal anti-inflammatory drugs (NSAIDs), and 14 patients (5%) were given a transdermal opioid analgesic patch for pain.

### Surgical treatment

25 patients (4%) with osteoporotic vertebral fractures underwent surgical treatment. Four of these 25 patients underwent vertebroplasty, 15 patients were treated by kyphoplasty, and the remaining 6 patients underwent posterior stabilization of the spine via an open or percutaneous approach. Table 2 provides an age-adjusted overview relating to the type of surgical procedure. 16 patients underwent surgical stabilization under general anesthesia, 9 patients received local anesthesia during their procedure. From the 9 patients with the local anesthesia, 7 patients were treated by vertebroplasty, the remaining two patients by kyphoplasty. In cases of posterior stabilization (n = 6), all patients required general anesthesia.

**Table 2:** Surgical procedure. Type of surgical procedure.

Age Group	Vertebroplasty (V)	Kyphoplasty (K)	Posterior Stabilization (PS)	Differences—V+K vs. PS (p-value)
50–59	0 (0%)	1 (4%)	1 (4%)	p = 0.101
60–69	0 (0%)	3 (12%)	3 (12%)	/
70–79	3 (12%)	6 (24%)	1 (4%)	p = 0.544
80–89	1 (4%)	5 (20%9	1 (4%)	p = 0.344
Over 90	0 (0%)	0 (0%)	0 (0%)	/
All Age Groups	4 (16%)	15 (60%)	6 (24%)	p = 0.043

### Complications / Undesired events

Complications or other undesired events were noted in 17 of 694 patients, representing an overall rate of 2%. The number of complications was distributed similarly among the various age groups. With regards to the type of treatment received, undesired events were seen notably more often in patients following posterior stabilization. This is demonstrated in Table 3.

**Table 3:** Complications. Distribution of complications.

Treatment	Number of Complication	Type of Complication
Conservative- Outpatient	2	1 Deterioration of Kyphosis 1 Paresthesia in both legs
Conservative- Inpatient	2	1 Deterioration of Kyphosis 1 Paresthesia of both legs
Vertebroplasty	4	1 Revision surgery 1 Paresthesia- thighs 1 Deterioration of kyphosis 1 starting myelopathy through posteriorly impinging material
Kyphoplasty	3	1 Paresthesia- leg unilateral 1 Narrowing of spinal canal through kyphoplasty material 1 Deterioration of kyphosis
Posterior Stabilization	6	1 Consecutive fracture of accompanying vertebral bodies 2 Revision surgeries 1 Cage tilted (but stable) 1 Deterioration of Kyphosis 1 Patient died (respiratory failure)

### Rehabilitation programs

Subsequent to treatment at our department, 33 patients (4%) were transferred to a health resort in order to undergo a professional rehabilitation program. 24 of these patients (3%) had undergone conservative therapy, one patient (0.1%) had received vertebroplasty, 6 patients (0.8%) had received kyphoplasty and 2 patients (0.3%) had undergone posterior stabilization.

### Duration of hospital stay

310 of the 694 patients (45%) were treated as inpatients. 285 of these inpatients underwent conservative management; the remaining 25 were treated surgically. The overall average duration of hospital stay was 10.8 days, (range: 1–32 days). Statistical analysis revealed no significant differences between genders, in any of the age groups. Duration of hospital stay was slightly higher in women following conservative treatment, though this was not statistically significant. We did not find any significant differences between conservative and surgical treatments with regards to hospital stay, either. 67 of 310 patients (22%) spent less than 3 days in hospital and were within the allowed time range for a flat rate payment. 63 of these patients were treated conservatively, 1 patient underwent vertebroplasty and 3 patients were treated with kyphoplasty. 224 patients (72%) could be dismissed between 3 and 14 days after admission and remained within the time frame suggested by the flat rate payment. 207 of these patients were treated conservatively, 2 were submitted for vertebroplasty, 11 were submitted for kyphoplasty and 4 patients received open surgery. 19 patients (6%) remained longer than 14 days, which was over the time allowed for a flat rate payment. 15 of these patients were treated conservatively, 1 was submitted for vertebroplasty, 1 was submitted for kyphoplasty and the remaining 2 patients were submitted for open surgery.

### Sick leaves

With regards to the number of sick leaves, only the age categories 50–59 and 60–69 were integrated for analysis, as the patients from the remaining age groups had already entered retirement. Ultimately, 212 patients (31%) were included for the assessment of the number of sick leaves, due to the fact that those patients had not yet retired as of the initiation of treatment. Statistical analysis revealed that, in reference to the conservative treatment of both inpatients and outpatients, the number of sick leaves was significantly higher in women than in men. When comparing treatment methods, we did not find any significant differences concerning the amount of sick leaves. Out of the 212 included patients, 127 (60%) had already been reintegrated into their working lives when treatment began, while 85 (40%) were either unemployed or identified as housewives.

### The need for professional domestic help

Out of 694 patients, 168 (24%) required professional domestic help. In 16 of these cases, this help had to be newly arranged and organized during their hospital stay. Concerning patient gender differences, statistical analysis revealed that the need for professional domestic help occurred significantly more often in women than in men. Notably though, for male patients, adequate domestic help was more often carried out by a female life partner (rather than the other way round). Statistical analysis also showed that the need for professional domestic help increased significantly in persons aged 70 and older.

### Prevention of osteoporosis and its related fractures

In 152 patients (22%), osteoporosis had been officially diagnosed utilizing axial DXA examination and T-scores < -2.5 hase been considered as verified Osteoporosis prior to presentation at the outpatient clinic. However, according to their medical histories, only 67 of those patients were put on an osteoporosis-specific medication–revealing 44% “adequate treatment rate” where osteoporosis and the primary prevention of osteoporotic fractures are concerned. In regards to the secondary prevention of osteoporosis and its associated fractures, 118 patients (17%) received an osteoporosis-specific medication from their general practitioner, as well as the recommendation for regular follow up controls (including T-score measurement). 92 of these patients were receiving supplemental vitamin D, calcium or bisphosphonates; the remaining 26 patients were receiving hormone replacement therapy or Raloxifen. In only 34 patients (5%) was a specialist for the treatment of osteoporosis consulted. Table 4 presents an age-adjusted and gender-related overview. Comparing gender, the prevention of osteoporosis and its related fractures, statistical analysis revealed that primary or secondary methods of prevention were seen significantly more often in women than in men. In only 4 men, osteoporosis was suspected to be a decisive factor for the associated fractures. Ultimately, osteoporosis-specific medication was recommended to 2 of these men, so as to avoid further osteoporosis-related fractures.

**Table 4:** Prevention. Previous treatment of osteoporosis and primary prevention as well as Secondary prevention of osteoporotic fractures and its associated fractures.

Age Group	Previously Diagnosed Osteoporosis		Intake of Specific Medication		Gender Differences (p-value)	Treatment / Prevention with General Practitioner		Treatment / Prevention with specialized Consultant		Gender Differences (p-value)
	female	male	female	male		female	male	female	male	
50–59	11	1	5	1	p = 0.124	3 (1%)	1 (0.5%)	11 (2%)	1 (0.5%)	p = 0.145
60–69	30	2	12	0	p = 0.010	18 (3%)	0	47 (9.5%)	1 (0.5%)	p = 0.042
70–79	46	1	26	0	p = 0.011	11 (2%)	1 (0.5%)	31 (6.5%)	0	p = 0.079
80–89	50	0	22	0	n.s.*	0	0	22 (4%)	0	n.s.*
Over 90	11	0	1	0	n.s.*	0	0	6 (1%)	0	n.s.*

### Treatment costs

The cost of outpatient treatment was calculated from both basic fees (i.e., a primary outpatient treatment fee of €80.57, and €53.72 for every consecutive treatment) and additional costs for obligatory X-ray examinations of vertebral segments (€80.24). For calculating costs incurred by the conservative treatment of inpatients or surgical stabilization, the procedure-oriented health care financing system was applied. Table 5 provides a detailed overview of the average per-patient costs of the various treatment methods. Statistical analysis did not reveal any significant differences when comparing treatment costs.

**Table 5:** Treatment costs. Average Treatment Costs and gender- differences per patient relating to the type of treatment.

Treatment	Average Value in Euros		Average Value in Euros	Gender Differneces (p value)
	female	male		
Conservative- Outpatient	€ 348.48	€ 348.48	€ 160.81 (Primary treatment with X-ray) € 133.95 (consecutive treatment with X-ray) € 53.72 (consecutive treatment without X-ray)	p = 0.212
Conservative- Inpatient	€ 3200.16	€ 3195.06	€ 3197.61 (without extra fees and deductions)	p = 0.168
Vertebroplasty	€ 8945,96	€ 8948.02	€ 8946.99 (without extra fees and deductions)	p = 0.189
Kyphoplasty	€ 8959,06	€ 8934,92	€ 8946.99 (without extra fees and deductions)	p = 0.120
Posterior Stabilization	€ 11440,45	€ 11444,19	€ 11442.32 (without extra fees and deductions)	p = 0.146

## Discussion

Several clinical trials have demonstrated that if an adequate therapy for osteoporosis and its associated fractures can be initiated early, bone mineral density (BMD) can thereby be increased by 5–15%, and the rates of vertebral fractures can be reduced by 40–70% [6–10]. This would ultimately lead to a lessened decrease in patient mobility, a smaller reduction in health-related quality of life, and lower morbidity and mortality rates. Osteoporotic vertebral body fractures are typically the result of minor (or in some cases, no) trauma, a fact which is also backed by the results of this study. Furthermore, the duration of elapsed time between trauma and presentation in the outpatient clinic is a good indicator of trauma severity. However, only 49% of the patients in our series reported to the outpatient clinic on the same day. In patients with high-energy spine trauma, the rate of same-day presentation in the outpatient clinic is usually over 95%. Concerning imaging procedures for the diagnosis of osteoporotic vertebral fractures, CT-scans and MRIs are increasingly being demanded in order to ensure adequate radiographic assessment. However, a lack of sufficient diagnostic measures supports the conjecture that the incidence of these injuries is commonly underreported, and that correct diagnosis is often missed. In our study, we noticed that computed tomography was performed in only 29% of cases in addition to the obligatory X-ray studies (so as to confirm the diagnosis); in only 4% of cases was magnetic resonance imaging performed in addition to X-rays. Thus, recent or older vertebral fractures with typical signs of osteoporosis could only be distinguished in 21% of all cases. Additionally, the extent of vertebral body deformity was only documented in 24% of the cases–also a result of inadequate diagnostic measures. With regards to the treatment of osteoporotic vertebral fractures, the vast majority of patients are treated non-operatively, either as in- or outpatients. Surgical therapy is reserved rather for patients with persistent pain symptoms or notable deformities of the affected vertebral bodies [11, 12]. Despite considerable costs, the number of minimally invasive procedures performed–such as percutaneous vertebroplasty and kyphoplasty–has significantly increased in the last years [13, 14]. In our series, 96% of patients received conservative treatment, and only 4% were admitted for surgery. The conservative approach was conducted within the framework of outpatient treatment in 55%, while 41% of patients were admitted to the outpatient clinic. The timeframe for hospital stay flat rate payments, as set by the “procedure-oriented financing of health care providers”, was met in 72% of cases; in 23% of cases it was undercut, and in 6% it was exceeded. Referring to outcomes after surgical or conservative therapy, treatment success is usually assessed by different variables–including pain reduction, recovery of mobility for clinical evaluation, and secondary sintering of the treated vertebral fracture for radiographic assessment [15, 16]. In our study, we were also not able to provide valid conclusions from the investigated parameters, as the number of long-term follow-up examinations was too low. Pain reduction in our patients was most frequently seen within the first 2 weeks after trauma. In the following course of check-up exams, no further significant alteration was observed. Hight reduction oft the vertebral body (secondary sintering) following treatment of the osteoporotic fractures was merely observed (5%), in most of these cases following conservative treatment. Undesired events and complications during or instantaneously after treatment were also rare (4%), with kyphoplasty exhibiting the lowest complication rate.

It is well known that the risk of vertebral fractures rises rapidly with age for both men and women. In the United States and in Europe, women are two to three times more likely than men to experience a vertebral fracture. In a population-based study, the age adjusted incidence of clinically diagnosed vertebral fracture was 145 per 100,000 person years in women compared to 73 per 100,000 person years in men [1, 17, 18]. Austria is basically on the same average.

Public Health research has detected the importance of osteoporosis and its related fractures as a burdening factor for the increase in health care expenditures [19, 20]. The socioeconomic burden of osteoporosis and its related fractures includes direct medical costs–such as those associated with acute and rehabilitative care–as well as indirect costs related to poor health status, to a prolonged or permanent use of professional domestic or social help, or to a relevant number of sick leaves [21]. For consideration of socio-economic aspects, we evaluated patient sick leaves, the need for professional domestic help, living situation and treatment costs, according to current literature dealing with similar topics [19, 20, 22]. Indirect costs (derived from various factors, such as the use of professional domestic help, the loss of manpower due to sick leaves, treatment of poor health status, etc.) are hardly calculable for each patient. Approximate estimations are commonly used in order to offer at least an overview of the situation [21]. Relating to the results in this series, fewer than 10% of patients (67) still belonged to the working population (i.e., integrated into the workforce) at the moment of treatment. The exact number of sick leaves, however, could not be deduced from patient histories. An approximate estimation, based on patients’ voluntarily provided information, revealed an average of 32.4 sick leave days, ranging from 11–68 days. With regards to living situation, 23% of all included patients reported to be living on their own. Domestic help as provided by a partner was only available to a small number of patients. The option of receiving care from children and other family members could not be exactly assessed from the data sets. Consequently, professional domestic help, which was ultimately documented in 24% of cases, played a central role in the care of patients suffering from osteoporosis–and might also play an important part in calculating indirect costs. In this context, statistical analysis within this study revealed significant gender differences, as women represented a more severe burden than men. This might be attributed to the fact that female patients had significantly less support from their life partner or from friends than the way round (i.e., the idea that women were “supposed to” care for their husbands).

Direct costs for acute medical care can be acquired easily in most nations, whereas direct costs for rehabilitative procedures are more complex and often regionally fluctuant. Referring to the results in our study, the costs incurred by conservatively treating outpatients with pain medication were the lowest–as was expected. In outpatients who were sent to physiotherapy or supported by an orthesis, the costs became insignificantly larger. However, costs accrued by the conservative treatment of inpatients were tremendously higher–in excess of twenty times higher (approximately €3.200), excluding extra fees and deductions. A further increase in total costs to the national health care system inevitably results from surgical treatment of osteoporotic vertebral fractures. The costs for the 2 current standard methods (kyphoplasty and vertebroplasty), performed as a single procedure, are in the thousands of Euros, depending upon the duration of hospital stay. However, for both vertebroplasty and kyphoplasty, the same amount is refunded to the health care providers. As material costs are much higher for the procedure of kyphoplasty, a financial disadvantage is created for this method from the perspective of health care providers. However, recent reports in the literature have purported superior outcomes after kyphoplasty (when compared to vertebroplasty) [23–25].

From a public health perspective, osteoporosis as a chronic disease is still underestimated, and often undertreated, by the medical care sector. Relating to osteoporotic vertebral fractures, the incidence of these injuries is underreported. The problem of undetected–and thus untreated–osteoporosis is also reflected in the results of this study. Knowledge and awareness of the disease was only present in 22% of osteoporosis patients within this series; even fewer of those patients were supplied with adequate medications, representing a rate of primary prevention of osteoporotic fractures of less than 10%. In terms of secondary prevention of osteoporosis-associated fractures, the efforts within this study group were even more discouraging. Only 17% of the patients received an adequate osteoporosis medication in accordance with the general practitioner as well as the recommendation for regular follow up controls–and in just 5% of cases was a specialist for the treatment of osteoporosis consulted. In this context, statistical analysis also showed significant gender related differences, as preventive measures for men were more or less completely neglected by the treating clinicians. In only 2% of male patients with typical osteoporotic fractures in this series was osteoporosis suspected to be a decisive factor for these associated injuries. In women, at least 23% were destined to receive secondary preventative measures so as to avoid further osteoporotic fractures.

## Conclusion

Specifically concerning primary and secondary prevention, the results of this study demonstrate a regrettably low rate of adequate treatment after diagnosis of osteoporosis and its associated fractures. Moreover, the efforts and expenditures within the medical care sector were diversified, whereas the collaboration with the Public Health sector was rather negligible. In accordance with former studies, significant gender differences–to the detriment of the female population–could be determined. These differences, however, relate to prevention of osteoporosis and its associated fractures. To prospectively avoid complications and subsequent cost increases, a higher awareness of the need for prevention, early diagnosis and adequate treatment of osteoporosis and its related fractures should be considered.
